# Mitochondria As Sources and Targets of Methane

**DOI:** 10.3389/fmed.2017.00195

**Published:** 2017-11-13

**Authors:** András Tamás Mészáros, Ágnes Lilla Szilágyi, László Juhász, Eszter Tuboly, Dániel Érces, Gabriella Varga, Petra Hartmann

**Affiliations:** ^1^Institute of Surgical Research, University of Szeged, Szeged, Hungary

**Keywords:** methane, review, mitochondrion, apoptosis, gasotransmitter

## Abstract

This review summarizes the current knowledge on the role of mitochondria in the context of hypoxic cell biology, while providing evidence of how these mechanisms are modulated by methane (CH_4_). Recent studies have unambiguously confirmed CH_4_ bioactivity in various *in vitro* and *in vivo* experimental models and established the possibility that CH_4_ can affect many aspects of mitochondrial physiology. To date, no specific binding of CH_4_ to any enzymes or receptors have been reported, and it is probable that many of its effects are related to physico-chemical properties of the non-polar molecule. (i) Mitochondria themselves can be sources of endogenous CH_4_ generation under oxido-reductive stress conditions; chemical inhibition of the mitochondrial electron transport chain with site-specific inhibitors leads to increased formation of CH_4_ in eukaryote cells, in plants, and in animals. (ii) Conventionally believed as physiologically inert, studies cited in this review demonstrate that exogenous CH_4_ modulates key events of inflammation. The anti-apoptotic effects of exogenously administered CH_4_ are also recognized, and these properties also suggest that CH_4_-mediated intracellular signaling is closely associated with mitochondria. (iii) Mitochondrial substrate oxidation is coupled with the reduction of molecular oxygen, thus providing energy for cellular metabolism. Interestingly, recent *in vivo* studies have shown improved basal respiration and modulated mitochondrial oxidative phosphorylation by exogenous CH_4_. Overall, these data suggest that CH_4_ liberation and effectiveness in eukaryotes are both linked to hypoxic events and redox regulation and support the notion that CH_4_ has therapeutic roles in mammalian pathophysiologies.

## Introduction

Methane (CH_4_) is a small omnipresent molecule, the simplest alkane, and the most abundant organic gas in the atmosphere (1). It can help control the amount of hydroxyl radicals and neutralizes ozone in the troposphere (2) and also plays a role in global warming. More importantly, CH_4_ can be synthesized biologically and several recent studies have revealed its bioactivity. In a pioneering study, radioactive ^14^C-labeled CH_4_ was administered to the systemic circulation of sheep, and radioactive carbon dioxide (CO_2_) was detected in the breath of animals. Since apart from burning, non-biological decomposition of CH_4_ would need high temperature (above 1,000°C) or catalysts, recovery of ^14^CO_2_ in the exhaled air suggest involvement of CH_4_ in the cellular metabolism. Unfortunately, the underlying mechanisms were never resolved (3). In line with the data of Dougherty and coworkers, in a pilot study both ^3^H- and ^14^C-labeled CH_4_ was administered to rats. The subsequent analyses revealed organ-dependent rates of retention and decomposition CH_4_ (4, 5), which was interpreted as involvement in the carbon metabolism.

In the past decades, several signaling cascades of small gaseous molecules, including nitric oxide (NO), carbon monoxide (CO), and hydrogen sulfide (H_2_S), have been recognized. These gases play vital roles in biological systems, and they are in the focus of research interest (5). Due to its characteristics, availability and effectiveness, CH_4_ became also a candidate gasotransmitter (6). The first reports about the protective effect of CH_4_ against oxidative stress and inflammation caused by ischemia and reperfusion (IR) (7, 8) was followed by several studies (9–12). Because of its wide-ranging protective effects in many diverse disease models, it was proposed that CH_4_ could be a new medical gas (13). Indeed, CH_4_ is intrinsically non-toxic, without any known side effects. However, before the use in human clinical settings, the specific mechanism of action needs to be elucidated.

The mitochondrion is feasible target. Mitochondria are specialized subcellular structures that power various physiological roles, such as energy production, reactive oxygen species (ROS) formation, calcium homeostasis, and intrinsic apoptosis, all of which may be targets of CH_4_ administration (3, 4). A previous review summarized the available findings on the biological role of CH_4_, and it was proposed that CH_4_ liberation is related to hypoxic events resulting in, or associated with mitochondrial dysfunction (6). Indeed, several studies have demonstrated that the effects of exogenously administered CH_4_ in IR injuries can be grouped around a typical triad, namely anti-inflammatory, anti-oxidative, and anti-apoptotic properties. Notably, all these changes are also associated with mitochondrial functions, probably *via* non-specific physico-chemical alterations of membranes.

Importantly, concentrations of exogenously applied CH_4_ are orders of magnitude higher than those reported for endogenous production (reviewed in Section “Endogenous CH_4_ Formation Is Associated with Mitochondrial Dysfunction”). Due to the low solubility of CH_4_ in the watery phase, the majority of the gas is exhaled while CH_4_ is enriched at biological membrane interfaces, leading to higher local concentrations. Therefore, no direct conclusion can be made about the role of CH_4_ as a messenger only based on studies with CH_4_ treatment.

Overall, this review summarizes the effects of CH_4_ on mitochondria along with the current knowledge and the best available evidences on the possible mode of action. First, mitochondria are discussed as sources of endogenous CH_4_ generation under oxido-reductive stress conditions. Next, the consequences of exogenous CH_4_ supplementation are outlined: how it modulates key events of inflammation that are associated with mitochondrial functions. Thereafter, CH_4_-mediated intracellular signaling events are overviewed that are likely involved in cellular protection. Finally, the impact on the mitochondrial substrate oxidation in relationship with the anti-apoptotic effects is discussed.

## Endogenous CH_4_ Formation is Associated with Mitochondrial Dysfunction

Mammalian methanogenesis has been considered an exclusive attribute of methanogenic *Archaea*, a group well distinguished from bacteria and eukaryotes. Nevertheless, to date, a number of studies have demonstrated the generation of non-bacterial CH_4_ also in aerobic living systems, and it has also been proposed that the CH_4_-producing phenomenon can be linked to the loss of the redox homeostasis.

It was shown in 2003 that hypoxia could lead to the generation of measurable amounts of non-bacterial CH_4_ in isolated liver mitochondria (14). Increasingly, high amounts of CH_4_ (between 0 and 2.3 nmol/mg protein) were generated after the addition of ascorbic acid and 1–100 mM hydrogen peroxide (H_2_O_2_), and the formation of CH_4_ was related linearly to the quantity of mitochondria incubated. A breakthrough came when Keppler and colleagues (15) provided direct evidence of CH_4_ generation in multi-cellular organisms under aerobic conditions. This key paper was followed by many studies that either supported or disagreed with the initial findings (16–19) where the common denominator was likely to be mitochondrial dysfunction. More importantly, it has been shown that oxido-reductive stress elicits aerobic CH_4_ emission in plants (20).

Interestingly, in 2008, a study by Ghyczy et al. (21) demonstrated aerobic CH_4_ emission in cultured endothelial cells exposed to hypoxia and metabolic distress. Mitochondrial dysfunction in this setting led to significant CH_4_ generation (~2–23 nmol/mg mitochondrial protein), depending on the nature and intensity of the metabolic distress, and a similarly high and dose-dependent CH_4_ generation was detected after ROS attack in the Udenfriend reaction.

Furthermore, it has been shown that the CH_4_-producing phenomenon can be mimicked by the administration of sodium azide (NaN_3_), a compound known to disrupt mitochondrial electron transport flow by specifically binding to cytochrome *c* (Cyt *c*) oxidase. In this *in vivo* study, the whole-body CH_4_ production profile was determined in unrestrained animals after chronic NaN_3_ administration (22). In this scenario, the stress-related methanogenic capacity of the rats was revealed in animals treated with antibiotics to eradicate the CH_4_-producing intestinal flora (22). In a model of transient mitochondrial distress, the CH_4_ generation of rats and healthy human volunteers was evaluated before and after excessive ethanol intake, and significant CH_4_ production was demonstrated in both species (23). The phenomenon was again independent from the activity of methanogenic prokaryotes (23).

The CH_4_-generating capacity of NaN_3_ administration may also be associated with the generation of ROS (24, 25). It has been hypothesized that electrophylic methyl groups of biomolecules such as the phosphatidylcholine molecule might be carbon precursors (14, 18) and a potential source of CH_4_ liberation. Nevertheless, the underlying mechanism is complicated by the various oxido-reductive stress answers of the mitochondria, e.g., throughout DNA methylation patterns and therefore gene expression changes. Such conditions can both affect gene expressions and activity of *S*-adenosylhomocysteine hydrolase (26) and may change the methionine metabolism, where CH_4_ may be liberated as an intermediate compound. Recently, Althoff et al. (27) presented a novel chemical reaction that readily forms CH_4_ from organosulphur compounds such as methionine, under highly oxidative conditions, ambient atmospheric pressure, and temperature. In this reaction, methyl sulfides are oxidized to the corresponding sulphoxides by a ferryl species, then, in the next phase, demethylation of the sulfoxide *via* homolytic bond cleavage leads to CH_4_ formation (27).

## Novel Signaling Pathways Involved in CH_4_-Mediated Nuclear and Mitochondrial Effects

CH_4_ might alter the pattern of the activation of various signal transduction pathways and *vice versa*, the well-described triple effect (i.e., anti-inflammatory, antioxidant, and anti-apoptotic) of CH_4_ may influence the upregulation and downregulation of cellular signaling cascades. A direct link between exogenously administered CH_4_ and signaling targets have been reported recently (28, 29); however, a crosstalk with other bioactive gases and pathways cannot be excluded. Furthermore, no specific binding of CH_4_ to any enzymes or receptors have been reported to date, and it is highly probable that many (if not all) of its effects are related to physico-chemical properties of the non-polar molecule.

**Figure 1:**
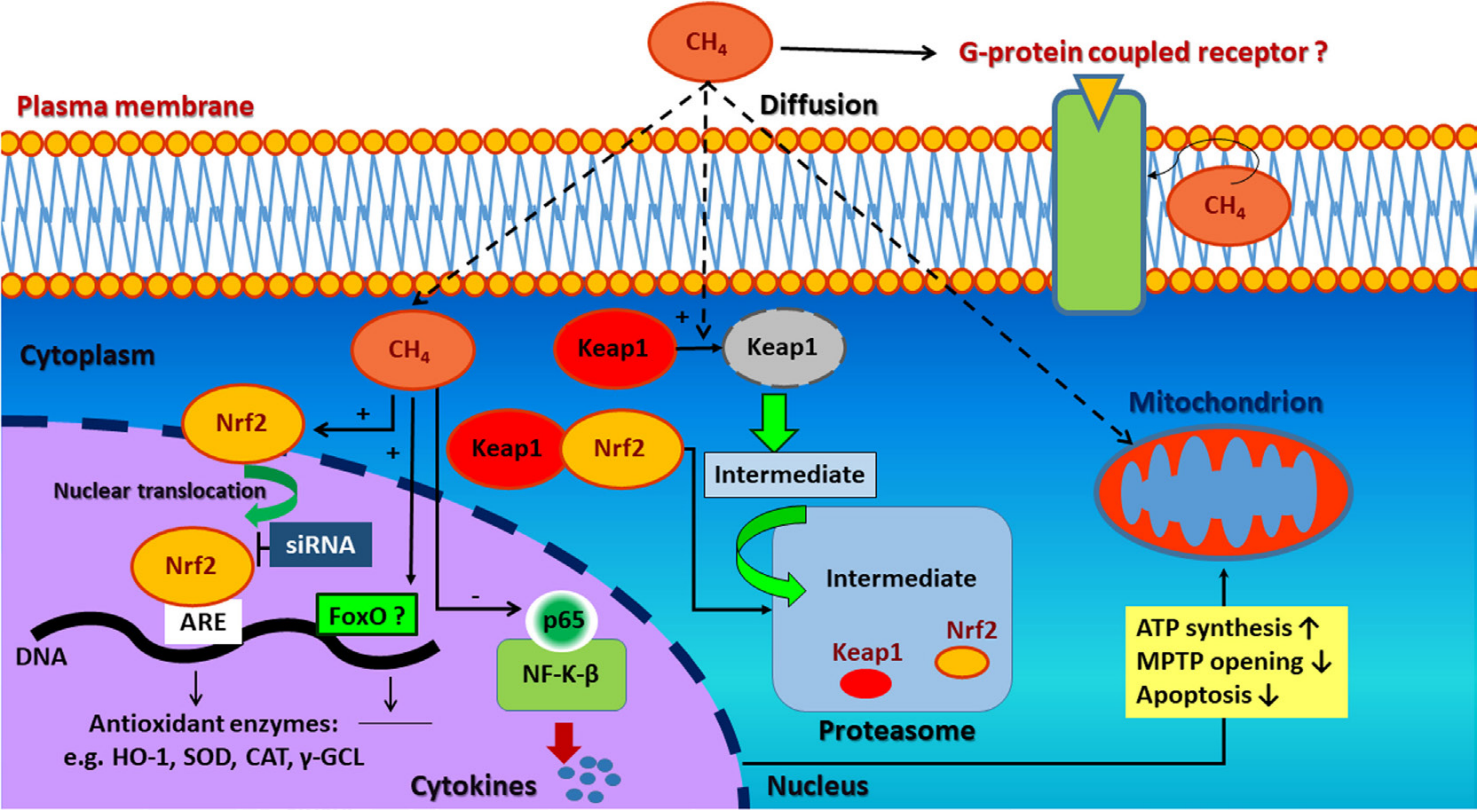
Possible signaling pathways involved in the antioxidant, anti-apoptotic, and anti-inflammatory effect of methane. CH_4_ may induce Nrf2/ARE-mediated activation of antioxidant and detoxifying enzymes. These attenuate the excessive production of reactive oxygen species (ROS) resulting preserved mitochondrial function as well as anti-inflammatory and anti-apoptotic effects. Second, complementary antioxidant pathways (e.g., FoxO) are also hypothesized to be activated. The non-polar nature of CH_4_ may influence cell membrane permeability and ion channel function-related signal transductionas well. Nrf2, nuclear factor erythroid 2-related factor 2; ARE, antioxidant response element; HO-1, hem oxygenase-1; SOD, superoxide dismutase; CAT, catalase; γ-GCL, γ-glutamyl cysteine ligase; siRNA, small-interfering RNA; FoxO, forkhead box transcription factor class O; CH_4_, methane; Keap1, kelch-like ECH associating protein 1; NF-κB, nuclear factor kappa-light-chain-enhancer of activated B cells; p65, transcription factor p65.

In a recent study by Wang et al. (29, 30), a supersaturated (~1.5 mmol/l) CH_4_-enriched saline solution was administered in a rat model of IR injury. High tissue concentrations of CH_4_ (between 90 and 145 µmol/g) were achieved, which lead to increased expression of Nrf2 (also known as nuclear factor erythroid 2-related factor 2). Nrf2 undoubtedly plays a central role in the activation of antioxidant defense system in most living organisms. It is now well characterized that Nrf2 translocates and binds to the antioxidant response element (ARE) forming a complex in the nucleus and induces the expression genes antioxidant and detoxifying enzymes (Figure 1) (31, 32). A negative regulator of Nrf2–ARE pathway is the kelch-like ECH associating protein 1 (Keap1), which forms a cytoplasmic complex with Nrf2 and inhibits its translocation to the nucleus under basal conditions (33).

Wang and coworkers (29, 30) have found that CH_4_ administration enhances Nrf2 expression at both the mRNA and protein levels. In this study, marked elevations in Nrf2 mRNA and protein levels were found while the regulatory Keap1 protein was degraded in a time dependent manner. Besides, activation of phosphatidylinositol 3-kinase–Akt pathway, an indirect mechanism involved in the synergistic activation of Nrf2–ARE oxidative stress response, may also play a role in CH_4_ action (28, 34). As a result of CH_4_-upregulated Nrf2/ARE signaling, activated downstream enzymes [e.g., hem oxygenase-1, superoxide dismutase (SOD), catalase, and γ-glutamyl cysteine ligase] attenuate the excessive production of ROS and result in preserved mitochondrial function (35) as well as anti-inflammatory (36–38) and anti-apoptotic effects (39, 40).

Among several transduction pathways, the forkhead box transcription factor class O (FoxO)-related antioxidant enzyme induction seems to be a novel candidate in the protective effects of CH_4_ (41). Although FoxO in cooperation with tumor suppressor p53 (42) regulates cell cycle arrest, it is also responsible for ROS elimination by promoting the expression of numerous antioxidant genes and detoxifying enzymes (43); such as SOD, catalase, glutathione peroxidase 2, glutathione-*S*-transferase, and a sulfiredoxin (42). Moreover, it may influence mitochondrial homeostasis (44) through the modulation a serine/threonine-protein kinase, PTEN-induced putative kinase, thereby contributing to cell survival. Nevertheless, in the absence of data confirming that CH_4_ acts on FoxO pathway (45) in animal disease models, this remains only a hypothesis.

## CH_4_-Mediated Actions on the Mitochondrial Electron Transport System (ETS)

CH_4_ has favorable distribution characteristics by penetrating membranes and diffusing into organelles including mitochondria (46); therefore, a potential effect of CH_4_ on the mitochondrial respiration has also been emerged. Upon exogenous administration, CH_4_ gets into the bloodstream through the alveoli of the lung and dissolves in the plasma. The cytoplasm/plasma solubility is near uniform for CH_4_; however, this ratio is much higher in hydrophobic substances such as the phospholipid biomembranes of mitochondria (47, 48). Of interest, the protein complexes of the mitochondrial respiratory chain are partially embedded in the inner mitochondrial membrane, exposing parts of them to the hydrophobic lipid bilayer, which makes them potential targets of the CH_4_.

The modulator effects of small gaseous molecules on mitochondrial respiration have been demonstrated in animal models of IR injury both *in vivo* and in hypoxic assays *in vitro* (11, 30, 49–51). Specifically, NO exerted protection through the activation of the mitochondrial K_ATP_ channel opening (50) and induced a sustained mitochondrial depolarization (49). H_2_S preserved mitochondrial membrane integrity and the complex I- and II-linked oxygen consumption rate (51). CH_4_ restored the ADP-dependent mitochondrial respiration, i.e., the oxydative phosphorylation (11, 30). During oxygen deprivation, the mitochondrial ETS is manifested lower rates of non-phosphorylating basal respiration. In addition, the ADP-dependent oxygen consumption, or in other words oxidative phosphorylation, is significantly depressed. In contrast, reperfusion conditions induce leakage of electrons from the ETS into the intermembranous space (52) that leads to increased ROS formation. These results suggest the sensitivity of both the resting state of ETS and the mitochondrial bioenergetic function to IR injury.

General effects of CH_4_ on IR-related mitochondrial dysfunction involve the restoration of the electron transport machinery of the inner mitochondrial membrane when oxygen concentration rises. In a study by Strifler et al. (11), mitochondria incubated in a medium with a gas phase containing 2.2% CH_4_–air mixture displayed a significantly improved leak respiration and increased recovery of oxidative phosphorylation. These effects of CH_4_ on the inner mitochondrial membrane can be explained with a hypothesis which presumes that CH_4_ dissolves in biological membranes thereby changing its oxidative stress-related rigidity (7).

In parallel with the general effects on the mitochondrial ETS, CH_4_ seems to exert site-specific action on protein complexes. Among the protein complexes of the mitochondrial ETS, complex IV (cytochrome c oxidase), which catalyzes the reduction of oxygen by ferrycytochrome to H_2_O, is target of the CH_4_ action. Indeed, endogenous CH_4_ generation occurs in plant mitochondria (53) and in mammalian cells after inhibition of complex IV by NaN_3_ (22). Meanwhile, exogenous CH_4_ administration in IR injury conditions resulted in reduced Cyt *c* release from the inner mitochondrial membrane and lower Cyt *c* oxidase activity in liver mitochondria (11).

Nonetheless, the above observations are all related to the *in vivo* effects of exogenous CH_4_ supplementation on mitochondria in oxido-reductive stress conditions. Interestingly, direct mitochondrial effects could not be shown when a 2.2% CH_4_–air mixture was administered isolated mitochondria *in vitro* (11). In other words, direct effect of CH_4_ on oxidative phosphorylation capacity and leak respiration in intact liver mitochondria cannot be shown *in vitro*. CH_4_ has relatively low solubility in watery phase, ranging from 37.2 to 19.1 mg/l between 0 and 35°C at 1 standard atmosphere. Therefore, high concentrations should be applied exogenously to overcome the limitation of low gas solubility. This means that approximately 1 mmol/l/min CH_4_ was usually applied as gas therapy (7) and 1.6 mmol/l in CH_4_-enriched saline (29). Consequently, CH_4_ concentrations in tissues upon CH_4_ treatment are certainly higher than the average endogenous levels. Still, due to the high affinity of Cyt *c* oxidase for oxygen (54), CH_4_ does not dysproportionate oxygen and thus does not limit mitochondrial respiration.

## CH_4_-Mediated Actions on Apoptosis

The anti-apoptotic effect is one of the most studied properties of CH_4_. Several studies demonstrated that CH_4_ modulates the intrinsic pathway of apoptosis (9–11, 29, 30, 55–58). The intrinsic pathway of apoptosis, also called the mitochondrial pathway owing to the essential involvement of mitochondria (59), which are not only the site where anti- and pro-apoptotic proteins interact but also the origins of high range of signal pathways that initiate the activation of caspases through various mechanisms (60). The large family of Bcl-2 homologs involves key proteins of intrinsic apoptosis described to organize the process (61). They can be divided into two classes: anti-apoptotic Bcl-2 family proteins (such as Bcl-XL, Bcl-w, Mcl-1, A1, Bcl-Rambo, Bcl-L10, and Bcl-G) and pro-apoptotic proteins [such as Bcl-2 associated X protein (Bax), Bak, and Bok] (62). The primary anti-apoptotic function of Bcl-2 is to block the release of Cyt *c*. In contrast, upon stress, pro-apoptotic members of the Bcl-2 family are activated (Bak or Bax) which leads to the mitochondrial outer membrane permeabilization and subsequent release of intermembrane space proteins such as Cyt *c*. Cyt *c* is attached to the inner mitochondrial membrane and shuttles electrons between complex III and complex IV. In response to membrane damage, it releases to the cytosol and activate the initiator procaspase-9 within the apoptotic protease-activating factor-1 (Apaf-1) apoptosome complex (63). Once activated, caspase-9 activates effector procaspase-3 which, in turn, can cleave various protein substrates, leading to the morphological and biochemical features of apoptosis (64).

**Figure 2:**
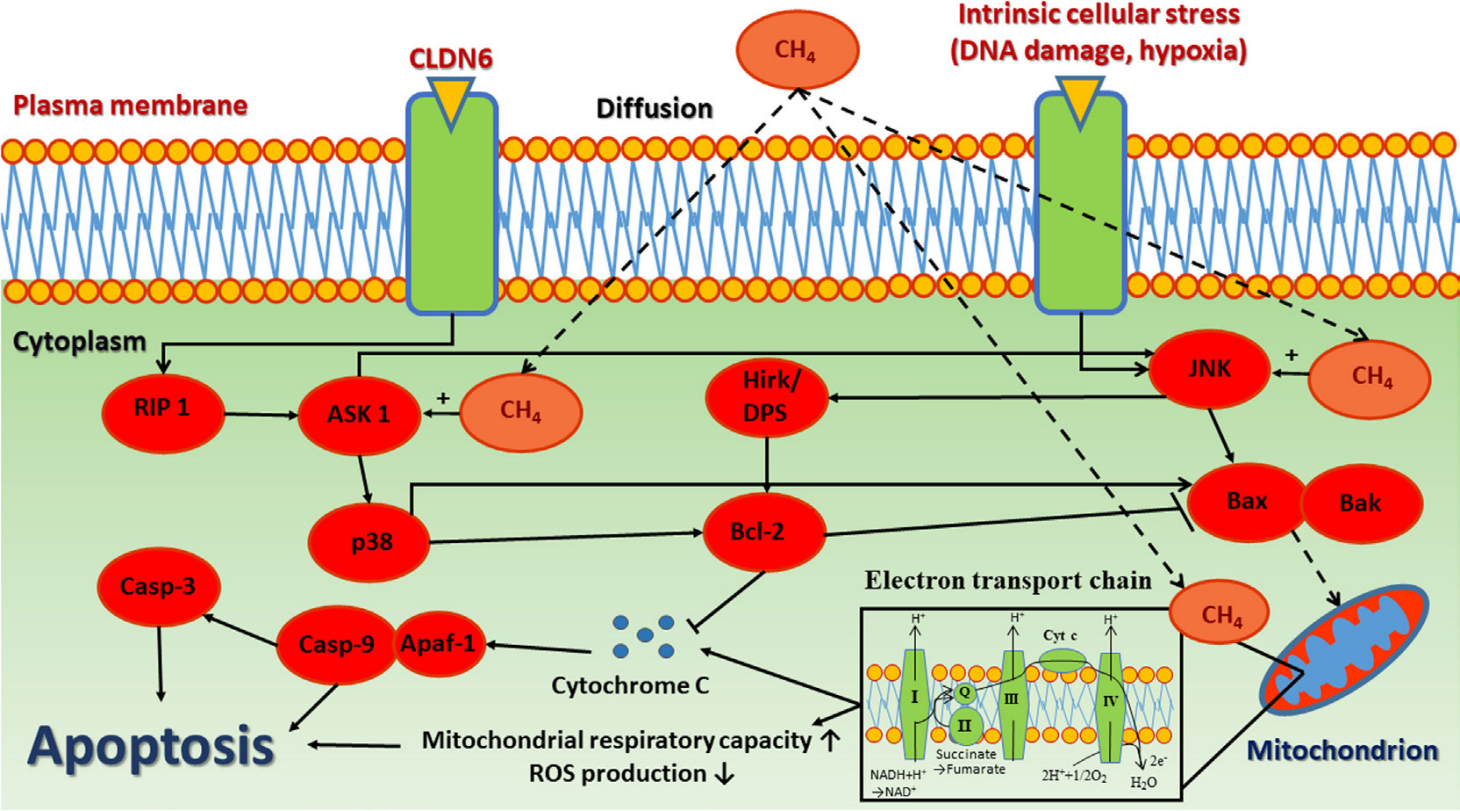
The anti-apoptotic effect of CH_4_. The release of cytochrome *c* and other inner mitochondrial membrane proteins are regulated by Bcl-2 family proteins through interplay between pro-apoptotic and anti-apoptotic proteins, which converge to Bax/Bak activation, thereby inducing mitochondrial outer membrane permeability. CH_4_, methane; CLDN6, claudin 6; RIP 1, receptor-interacting kinase 1; ASK-1, apoptosis signal-regulating kinase 1; p-38, mitogen-activated protein kinase; Casp-3, caspase 3; Casp-9, caspase 9; Apaf-1, apoptotic protease-activating factor 1; Hrk/DP5, harakiri gene; Bcl-2, B-cell lymphoma 2 regulation protein; JNK, c-Jun N-terminal kinase; BAX, Bcl-2 associated X protein; BAK, Bcl-2 homologous antagonist/killer.

In IR, CH_4_ supplementation may improve the level of key regulators of apoptosis, such as Bcl-2, Apaf-1, and caspases indirectly through an unknown pathway (10). Two recent studies of experimental retinal IR provided evidence for the effectiveness of CH_4_ treatment on upregulation of anti-apoptotic Bcl-2 proteins, while reduced cleaved caspase 3- and 9 quantities (10, 57). Likewise, in an experimental spinal cord IR scenario, CH_4_ was proven to significantly reduce the number of apoptotic cells (confirmed by TUNEL staining) and also the amount of cleaved caspase-3 and caspase-9 proteins and to attenuate cytosolic Cyt *c* release (29). In addition, CH_4_-rich fluids significantly reduced the expression of cleaved caspase-3 and also decreased the apoptotic cell number in rodents exposed to LPS-induced acute lung injury (58). In a hepatic IR model, CH_4_ inhalation could effectively attenuate the apoptosis-linked morphological changes in the liver and the TUNEL positivity of hepatocytes (11). Similarly, caspase-3 expression was attenuated with CH_4_-rich saline treatment which substantiated this finding (56). Moreover, CH_4_-rich saline has pronounced neuroprotective effect in diabetic retinopathy in streptozotocin-induced diabetes, possibly by upregulating those cell cycle related microRNAs, which contribute to post-transcriptional regulation (65). A possible indirect way how CH_4_ supplementation modulates apoptosis is to reduce the level of mitochondrial ROS formation. ROS and their by-products can oxidize the reduced thioredoxin-apoptosis signal-regulating kinase 1 complex, then activate apoptosis signal-regulating kinase and its downstream stress signaling targets, such as c-Jun NH(2)-terminal kinase (66). Another plausible explanation for the anti-apoptotic effect of CH_4_ is reducing Cyt *c* release from the inner membrane, which has already been demonstrated in hepatic, myocardial, and spinal cord IR as well (9, 11, 29) (Figure 2).

## Perspectives and Concluding Statements

In the human body, many gases are biologically active. Signaling roles were demonstrated for NO, CO, and H_2_S, and it has become clear that gaseous mediators form complex intracellular pathways and regulate numerous physiological processes, separately, or more often, in antagonistic or synergistic ways. CH_4_ is a small, less reactive gas molecule, having a close symbiosis with bioactive gases in the intracellular spaces. The effects of exogenous CH_4_ were clearly illustrated in detail in various tissues under different conditions. Of particular interest is that the recognized biological effects of CH_4_ are not cell- or tissue specific, and an increased input may result in anti-inflammatory changes in cells and tissues. In this regard, it is tempting to speculate on a much broader, controller role for CH_4_ in acute and chronic oxido-reductive stress conditions.

## Author Contributions

AM and PH designed and developed the concept of the manuscript. AS, LJ, ET, DÉ, and GV wrote the manuscript. ÁS prepared Figure 2. LJ prepared Figure 1. PH supervised and edited the manuscript. All authors discussed and commented on the manuscript at all stages.

## Conflict of Interest Statement

The authors declare that the research was conducted in the absence of any commercial or financial relationships that could be construed as a potential conflict of interest. The reviewer UA and handling editor declared their shared affiliation.
